# Borderline tumours of the ovary, current controversies regarding their diagnosis and treatment

**DOI:** 10.3332/ecancer.2013.379

**Published:** 2013-12-17

**Authors:** María Guadalupe Patrono, Lucas Minig, Ivan Diaz-Padilla, Nuria Romero, Juan Francisco Rodriguez Moreno, Jesus Garcia-Donas

**Affiliations:** 1 Gynaecology Oncology Programme, Clara Campal Comprehensive Cancer Centre, HM Hospitals, Madrid 28050, Spain; 2 Gynaecology Oncology Programme, Medical Oncology, Comprehensive Oncology Centre Clara Campal, HM Hospitals, Madrid 28050, Spain.

**Keywords:** borderline ovarian tumour, conservative treatment of fertility, gynaecological cancer, ovarian cancer surgery

## Abstract

Borderline ovarian tumours generally affect women of reproductive age. The positive prognosis is related to the fact that over 80% of cases are diagnosed at an early stage of the disease. Although radical surgery is the standard of care for this disease, fertility-sparing surgery can be performed in selected cases. Since it was first described in 1929, the knowledge of the molecular and histologic characteristics has been significantly improved. In this review, advances in the clinical behaviour, pathologic characteristics, prognostics factors, and different strategies of treatment are discussed.

## Introduction

Epithelial tumours of the ovary can be either benign (cystadenomas) or malignant (cystadenocarcinomas). However, there is an intermediate state of epithelial tumours of the ovary called ‘borderline tumours’. Neither the oncological behaviour of this intermediate group of tumours nor the histological changes of the cells of the ovarian epithelium meet the specific criteria of benignity or malignancy. In 1973, the International Federation of Gynaecology and Obstetrics (FIGO) gave this group of ovarian tumours a ‘low malignant potential’ [1], and since then, the World Health Organization (WHO) has called them borderline ovarian tumours (BOTs) [2].

They are tumours that usually occur during the third to fourth decade of women’s lives and are diagnosed as being limited to the ovary in 80% of cases. Because of this, their biological–oncological behaviour is very good, with an overall survival rate of ten years for 90% of those in the initial stages [3] and 60–70% of those in the advanced stages [4, 5].

While the recommended treatment is a hysterectomy with double adnexectomy, this frequently raises the clinical dilemma of diagnosis in women who have not yet given birth. Here, the conservative treatment of fertility may be a safe possibility in selected cases. The effectiveness of the chemotherapy is limited due to the slow rate of growth of the altered cells, meaning its use is limited and not advised. This chapter will describe the main histological features and clinical behaviour, and the different therapeutic options at the time of both the diagnosis and the recurrences of BOTs.

## Epidemiology and risk factors

BOTs comprise 15–20% of epithelial tumours [6]. Unlike invasive ovarian cancer, these tumours are diagnosed in the early stages in 80% of cases [7]. The average age at which such tumours are diagnosed is 40 years, but almost 30% of women with them are diagnosed before the age of 40 [8, 9].

Ritman *et al *[10] assessed the risk factors for BOTs in a case control study using the regional registry of tumours in Sweden. They randomly assigned 3899 control patients from the population register of all the residents in the country. The results showed that the women who had given birth more than once had a lower risk of developing borderline tumours compared with those women who had not given birth at all [odds ratio (OR): 0.44 (confidence interval (CI): 0.26–0.75) for serous tumours and 0.63 (CI: 0.34–1.19) for the mucinous tumours]. The authors found, in contrast, that breast-feeding served as a protective factor. This is similar to what has been observed for ovarian cancer. However, unlike the latter, the use of oral contraceptives was not a protective factor against the development of BOTs [OR: 1.4 (CI: 0.87–2.26)]. Thus, the authors suggested that this finding could support the concept that BOTs would represent a distinct disease to that of invasive ovarian cancer [10].

## Classification and histological characteristics

BOT of the ovary is characterised histologically by the presence of epithelial cells with nuclear atypia and mitotic activity in 10% or more of the tumour but without ovarian stromal invasion [8, 11]. The histological subtypes of epithelial BOTs can be serous, mucinous, endometrioid, clear, and transition cells (Brenner). The first two variants include 95% of the total [9].

### Serous BOTs

Serous tumours represent 65% of all the BOTs [9]; they are unilateral in 70% of cases, and 30% of them can occur with peritoneal implants. In turn, it is important to note that almost 30% of these implants have microscopic characteristics of stromal invasion [12] The implants with and without invasion are described in this way, reducing the ten-year survival from 95% to 60%, respectively [13].

The relationship of serous BOT as a precursor of invasive carcinoma has been extensively studied in recent years [14], suggesting two morphology-pathogenic tracks of epithelial ovarian tumours, based on their molecular differences and on their clinical behavioural–biological differences [15]. In this way, the serous tumours of type 1 would have a slow growth, generally limited to the ovary at the time of diagnosis, and would be developed from well-established precursor lesions from the cystadenoma, serous BOT until the micropapillary carcinoma low-grade serous. Genetically, they are characterised by mutations in the track of the KRAS, BRAF, PTEN, and A-catenin [15].

Reinforcing this theory, a study published by Shvartsman *et al *[16] found that serous BOTs often coexist with low-grade serous ovarian carcinomas. The purpose of the study was to compare the oncological outcomes of patients with low-grade serous carcinoma stage II/IV (group 1), with patients with relapsing BOT as low-grade serous carcinoma (group 2). The time free of the disease was studied from its diagnosis in group 1 and from the first relapse in group 2. We identified 112 patients in group 1 and 41 in group 2. There were no statistically significant differences between the two groups in average age (42.7 versus 45.4 years, *p *= 0.37), progression-free survival (19.5 months versus 25, *p* = 0.92), nor in the overall survival (81.8 versus 82.8 months, *p* = 0.84) [16].

Ovarian tumours of type II, in contrast, are fast-growing without precursor lesions and with a high degree of aggressiveness [15]. They manifest themselves as high-grade ovarian carcinomas, with extensive peritoneal dissemination and extra-abdominal disease and include the high-grade serous carcinomas, malignant mixed mesodermal tumours (carcinosarcomas), and undifferentiated carcinomas [15]. This group of tumours has a high level of genetic instability and is characterised by the mutation of the P53 gene and the overexpression of the genes of HLA-G, HER2, and AKT [17]. Most of the malignant epithelial tumours of the ovary belong to this type II.

### Borderline ovarian mucinous tumours

Mucinous BOTs represent 32% of all epithelial BOTs [7]. They are divided into two histologic subtypes: the intestinal (90%) and the Müllerian (endocervical type). In contrast to the serous BOT, the mucinous subtype is associated more rarely with peritoneal implants [9]. Like the serous tumours, the survival of women with stage I is 100%, while that in advanced stages is only 50% [9].

Following the type I morphology-pathogenic pathway previously described for the serous tumours, the mucinous BOT usually reaches a large size, tends to be unilateral, and can coexist with areas of mucinous cystadenoma or low-grade invasive carcinoma [14].

## Diagnosis

Clinical differentiation between borderline ovarian cysts, benign or malignant, is difficult. The majority of patients show an asymptomatic adnexal mass, in the annual gynaecological exam as an incidental finding in a gynaecological ultrasound. Of all forms, the most frequent symptoms are those of any type of adnexal mass and include abdominal pain, changes in intestinal transit, pelvic pain, and dyspareunia among others. The ability to manifest itself with abdominal distension because of ascites is less likely than in cases of ovarian cancer.

Vine *et al *[18] evaluated the symptoms and their duration before the diagnosis of invasive cancer or BOT. The authors observed that patients with BOT were twice as likely to be asymptomatic at diagnosis. At the same time, women with BOT were two times more likely to be diagnosed during a routine examination. Among the women with symptoms, those with BOT had a greater duration of symptoms than women with ovarian cancer (six versus four months). These aspects of the clinical presentation of BOT are probably a reflection of the more indolent nature of these tumours.

The vaginal ultrasound is the first step in the evaluation of patients with an adnexal mass. Exacoustos *et al *[19] determined that the presence of papillae within the cyst was the most common finding in the BOT. However, neither the sonographic features nor the papillae are ultrasound markers of high sensitivity. A Japanese study [20] also showed the usefulness of employing magnetic resonance imaging (MRI) to distinguish between BOTs and other conditions that are invasive to the ovary.

The tumour marker CA-125 may be high in more than half of the patients with BOT [8]. Engelen *et al *[21] assessed the level of the CA-125 preoperatively, which was found to be high in 8 of 33 (24%) patients, levels of CEA in 3 of 32 (9%), and levels of CA 19-9 in 11 of 24 (46%) cases. In patients with mucinous BOT, CA 19-9 was most frequently high (8/14, 57%) when compared with the CA-125 (3/20, 15%) (*p *= 0.02) or the CEA (2/18, 11%) (*p *= 0.02). Ayhan *et al *[22] found that the positivity of the CA-125 in the BOT serous group was statistically greater than in the group of BOT mucinous, whereas positivity for CA 19-9 and CEA in the mucinous histology was significantly greater than those of the serous tumours after analysing 60 patients.

Kolwijck *et al *[23] found that preoperative serum levels of CA-125 were significantly higher in patients with advanced stages (median 181 IU/ml; reaching values of I413 U/ml) compared with patients with initial stage (median 28 IU/ml; reaching values of 1123 IU/ml). The median in patients with serous histology was 59 IU/ml compared with the mucinous histology, which was 25 IU/ml.

Other diagnostic methods such as MRI and positron emission tomography-computed tomography (PET-CT) are usually reserved for selected cases since the sonographic features of the adnexal mass, coupled with the value of the tumour marker CA-125, are usually sufficient to indicate diagnostic or therapeutic surgery. Computed tomography (CT) is useful in the case of an adnexal mass with suspected BOT or malignancy. The objective is to detect intra-abdominal presence of disease [24].

In any case, the definitive diagnosis of the adnexal mass is made by an intraoperative histological study. In this sense, many studies have evaluated the accuracy of the histological diagnosis of the adnexal mass [25, 26]. As a result, the diagnosis is not often easy, mainly in large and mucinous tumours [25, 26]. The intraoperative diagnosis has proven to be reliable in discriminating between a benign and a malignant mass in a BOT (overdiagnosis less than 10%). However, a subdiagnosis of 25–30 % has been shown in differentiating a BOT from a malignant tumour [27, 28].

The clinical consequence of this limit is the need to re-operate to surgically stage the cases diagnosed as ovarian cancer in the final histological analysis that had been BOT in the intraoperative diagnosis.

## Treatment

**Figure 1:**
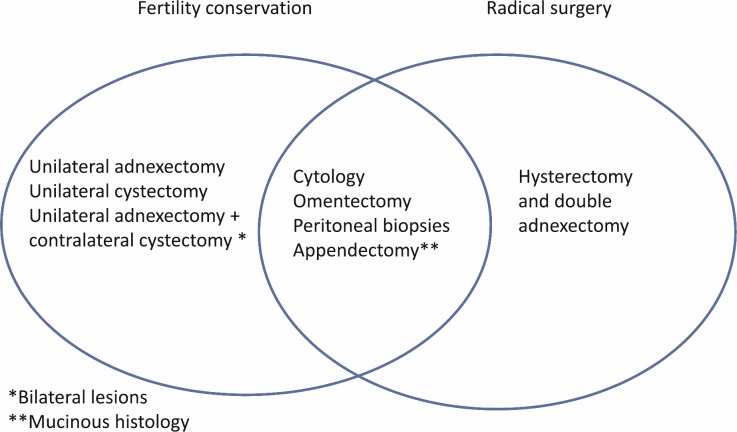
Surgical procedures in patients with borderline tumor with and without the desire to preserve fertility.

Surgery is the initial treatment for BOTs. Its principle is the same as that in invasive cancer, to remove the whole of the disease that is macroscopically visible. The recommended surgical staging includes having a hysterectomy, bilateral salpingo-oophorectomy, omentectomy, multiple biopsies, and peritoneal cytology. In the case of mucinous BOTs, an appendectomy should also be performed (Figure 1). The role of a lymphadenectomy has been extensively discussed in recent years [29–32]. However, according to the results of multiple studies, a pelvic and aortic lymphadenectomy does not improve the disease-free time or overall survival rate for women with BOT [29–33].

In contrast, the diagnosis may also be made at the time of the removal of a seemingly benign ovarian cyst. In that case, the dilemma is whether the patient should or should not be re-operated on, with the objective of completing the surgical staging and of making a careful inspection of the entire abdominal and pelvic cavity, to detect the presence of possible peritoneal implants. According to the data from the literature, this must be done mainly in the serous subtypes [33]. However, the majority of authors recommend this routinely, regardless of the histological subtype of the BOT [34, 35].

## Staging of borderline tumours of the ovary

The FIGO and the American Joint Committee on Cancer (AJCC) have designated stages to define ovarian tumours of a low malignant potential. The FIGO system as seen below is the most frequently used (Table 1) [36, 37].

## Prognostic factors

In patients with BOTs in advanced stages (II–IV), prognostic factors include age at the time of diagnosis, FIGO stage, residual disease following surgery, type of peritoneal implants (with or without invasion), presence of microinvasion in the ovarian tumour, micropapillary pattern, and the CA-125 value [38].

Approximately 30% of patients with serous BOTs have peritoneal implants at the time of diagnosis. Of these, 30% are invasive implants [39]. Morice *et al *[40] evaluated 80 patients with serous BOTs in FIGO stages II–III, 65 of whom had non-invasive implants, and 15 invasive implants. They found that the only prognostic factor for patients with advanced stage was the presence of invasive peritoneal implants. Reinforcing this idea, another study found that, although the overall survival rate of seven years for BOTs in advanced stages is 95% [41], in the presence of invasive implants, it can drop to 33% [42]. The extent of residual disease is another important prognostic factor. In a series of 168 patients with serous BOTs (FIGO stage II and III), the survival rate of five years was 75%, 76%, and 56% (*p *< 0.02) in patients without residual disease, with residual disease 1–20 mm or more than 20 mm, respectively [43]. In addition to the therapeutic potential, complete elimination of peritoneal implants allows for complete histological analysis of the disease [11].

## Adjuvant chemotherapy

Stage I BOTs do not need adjuvant treatment. A retrospective study from the Gynaecologic Oncology Group (GOG) analysed 988 adequately staged patients with stage I BOTs who did not receive adjuvant treatment and observed a mortality rate of 0.7% at five years [44].

In contrast, the role of adjuvant therapy in women with advanced stage BOTs is debatable. A meta-analysis of from Cochrane on BOTs concluded that current evidence does not show any benefits in the use of adjuvant therapy, whether it is chemotherapy or radiotherapy, independently of the stage or tumour histology [45].

Trope *et al *[46], after a review of four randomised studies, concluded that adjuvant treatment in patients with stage I of the disease, significantly increases intestinal, neurological, and haematological toxicity, without therapeutic benefits. Nor did Sutton *et al *[47] find benefits in the use of chemotherapy in patients with stage III randomly assigned to receive treatment with cisplatin plus cyclophosphamide with or without doxorubicin in a prospective study from the GOG. Of the 32 patients selected after initial cytoreduction, 19 had residual disease less than 1 cm, and 13 remained without residual disease. Twenty (62.5%) received cisplatin plus cyclophosphamide and 12 cisplatin, cyclophosphamide, and doxorubicin as monotherapy; 75% of the patients received six or more cycles. A second exploration was done in 15 cases; only six were negative. However, with an average follow-up of 31.7 months (range 1–75), 31 patients remained alive without clinical evidence of disease.

## Conservative fertility treatment

Conservative fertility treatment, which consists of removing the entire disease but preserving the uterus and at least a part of an ovary, is especially important in women with BOT since nearly 30% of women are diagnosed before 40 years old, and many of them have not even met their expectations for reproduction [8, 9]. The conservative treatment with BOT consists of doing peritoneal cytology, infracolic omentectomy, peritoneal biopsies, and appendectomy in the case of mucinous BOTs (Figure 1).

Conservative surgery has been extensively evaluated in recent years. After analysing more than 2000 published cases, conservative fertility surgery is associated with a major risk of recurrence of the disease but has no impact on the overall survival rate [48–50].

The radicalism of conservative surgery, either ovarian cystectomy or unilateral oophorectomy, must be based on the extension of the disease and the presence of factors associated with a bad prognostic advising against conservative treatment [11]. These include the presence of microinvasion, a micropapillary pattern, and invasive peritoneal implants [51]. In contrast, based on the data from the literature, there do not seem to be contraindications for the use of drugs for ovarian stimulation in case of getting future pregnancies following the diagnosis and treatment of the disease [52, 53].

Another controversial point is whether the patients who have received conservative treatment of fertility must later receive radical surgical treatment with removal of the uterus and remaining ovary after meeting their reproduction desires, once they reach menopause or waiting on a possible disease relapse, independently of the age of the patient.

Given that there are no uniform criteria in that regard, possibly the most important factor of which to take note is the stage of the tumour at the time of diagnosis. So one could contemplate completing the staging at the time of recurrence or after having met the reproduction desires in patients with early or advanced stages, respectively [40, 51].

## Follow-up

Today there is no consensus with respect to the best way to follow-up with patients following the initial treatment for BOT for the early detection of recurrence. It seems reasonable that the sum of methods such as physical examination, CA-125 determination, and CT is a method of choice. Vaginal gynaecologic ultrasound is of vital importance in women who receive conservative treatment of fertility. It is important to know that the free time of disease for women with BOT who show a relapse is significantly major with respect to women with invasive ovarian carcinoma. Uzan *et al *[54] observed a time up until the recurrence of up to 31 months (range 4–242 months) in 162 patients with advanced stage serous BOT. Similarly, recurrences as far apart as 15 years following initial diagnosis have been described [55]. These data force the prolongation of the time of follow-up with said patients for many years following the diagnosis. In this way, the recommended follow-up must be every three months for the first two years, every six months from two to five years and must continue every year up to 15 years after the initial diagnosis [55].

Zanetta *et al *[56] analysed prospectively the follow-up of 164 women with stage I BOT who have undergone conservative surgery of fertility. The follow-up was done with a physical examination and vaginal ultrasound every three months for two years and every six months after that. The CA-125 determination was planned every six months in patients with a serous BOT. The authors concluded that the vaginal ultrasound is the most effective diagnosis technique in this group of patients. The clinical practice guide of the National Comprehensive Cancer Network (NCCN) [57] recommends follow-up with gynaecologic ultrasound; but in those patients with invasive implants the follow-up must be similar to that of ovarian cancer: CT of thorax-abdomen-pelvis and CA-125. Likewise, women who meet maternity expectations and were previously treated under conservative must receive the aforementioned standard surgical treatment [57].

With respect to hormonal contraception, the Centres for Disease Control and Prevention (CDC) classifies as category 1 (there is no restriction on the use of contraceptive methods) the use of combined contraceptive pills as well as progestins, patches, vaginal ring, copper intrauterine device and levonorgestrel, birth control methods for patients with ovarian cancer; hence the choice of contraceptive methods is not considered limited by the diagnosis of a BOT [58].

Something similar happened with the use of hormone replacement therapy (HRT): a Swiss study done by Mascarenhas *et al *[59] analysed the five-year survival rate in patients with BOT and invasives that received HRT. They did a prospective cohort study in which they included 799 women diagnosed with invasive cancer (*n *= 649) and BOT (*n *= 150) between the ages of 50 and 74 years in 1993–1995. Following five years of follow-up, 45% of patients with carcinoma and 93% of patients with BOT were alive. For women with BOT, there was no association between the use of HRT used before or after diagnosis and the survival rate. Nor was there any association for women with ovarian cancer.

## Treatment of relapses

Surgical treatment with the objective of maximum cytoreduction is the goal of treatment for relapsed BOTs [7, 34, 60]. This concept is equally valid for women following a conservative fertility pretreatment who want to do it again but with adequate counsel, accepted the major risk of relapse and with the concrete possibility of a strict follow-up [34, 61].

## Conclusion

BOTs constitute a group of epithelial tumours that can affect women of reproductive age who have excellent prognosis due to the low aggressiveness and the fact that they are largely diagnosed in initial stages. Conservative surgery of fertility is possible in select cases with disease limited to the ovary and when there is a strong desire to become a mother. Radical surgery of maximum cytoreduction is the goal of treatment in women with advanced or recurrent diseases. Residual disease following surgery and the presence of invasive peritoneal implants are the main prognostic factors. The benefits of adjuvant chemotherapy are limited by the fact that its use is still inadvisable. The follow-up of patients must be done up to 15 years following the initial diagnosis.

## Conflicts of interest

The authors have no conflicts of interest to declare.

## Figures and Tables

**Table 1.** FIGO staging of borderline ovarian tumours.

Stage	
I	Tumour limited to the ovary
Ia	Tumour limited to an ovary, absence of malignant cells in ascites, intact capsule without tumour extension on the ovarian surface
Ib	Tumour limited to both ovaries, absence of malignant cells in ascites, intact capsule without tumour extension on the ovarian surface
Ic^a^	Presence of tumour cells in ascites or peritoneal lavage, presence of tumour on the ovarian surface of one or both ovaries, broken capsule
II	Condition of one or both ovaries with pelvic extension
IIa	Extension and/or in utero metastasis and/or fallopian tubes
IIb	Extension to other pelvic tissues
IIc^a^	IIa or IIb with the presence of tumour cells in ascites or peritoneal lavage, presence of tumour on the ovarian surface of one or both ovaries, broken capsule
III	The tumour compromises one or both ovaries with histologically confirmed peritoneal implants outside of the pelvis and/or positive pelvic lymph nodes. Superficial hepatic metastasis corresponds with stage III. The tumour is limited to the true pelvis but with histologically confirmed malignant extension in the small intestine or the omentum
IIIa	Tumour limited to the pelvis with negative nodes, positive peritoneal implants, or extension to the small intestine or the mesentery
IIIb	Condition of one or both ovaries with histologically confirmed implants, positive peritoneal metastasis, no more than 2 cm in diameter, and the nodes are negative
IIIc	Peritoneal metastasis beyond the pelvis > 2 cm in diameter and/or positive regional lymph nodes
IV	Condition of one or both ovaries with distant metastases. Positive pleural effusion. Metastasis of the hepatic parenchyma

a To assess the impact in the diagnosis of stages Ic or IIc, it would help to know if the rupture of the capsule was spontaneous or caused by the surgeon and if the source of the malignant cells detected was in the peritoneal lavage or ascites.
